# Substanzkonsum und Nutzung von sozialen Medien, Computerspielen und Glücksspielen unter Auszubildenden an beruflichen Schulen

**DOI:** 10.1007/s00103-024-03854-0

**Published:** 2024-03-25

**Authors:** Kirsten Lochbühler, Monika Rossa, Christopher Ebert, Matthis Morgenstern, Nicolas Arnaud, Ludwig Kraus

**Affiliations:** 1 IFT Institut für Therapieforschung, Centre for Mental Health and Addiction Research, München, Deutschland; 2 Institut für Allgemeinmedizin, Klinikum der Universität München, LMU München, München, Deutschland; 3 IFT-Nord Institut für Therapie- und Gesundheitsforschung, Kiel, Deutschland; 4 Deutsches Zentrum für Suchtfragen des Kindes- und Jugendalters, Universitätsklinikum Hamburg-Eppendorf (UKE), Hamburg, Deutschland; 5 Department of Public Health Science, Centre for Social Research on Alcohol and Drugs, Stockholm University, Stockholm, Schweden; 6 Institute of Psychology, ELTE Eötvös Loránd University, Budapest, Ungarn; 7 Zentrum für Interdisziplinäre Suchtforschung, Universität Hamburg (ZIS), Klinik für Psychiatrie und Psychotherapie (W37), Universitätsklinikum Hamburg-Eppendorf, Hamburg, Deutschland

**Keywords:** Berufsschüler, Alkohol, Tabak, Cannabis, Problematischer Konsum, Prävention, Alcohol, Tobacco, Cannabis, Problematic use, Prevention

## Abstract

**Hintergrund:**

Das Ziel der vorliegenden Arbeit war die Erfassung der Verbreitung des (problematischen) Konsums von Alkohol, Tabak und Cannabis sowie der (problematischen) Nutzung sozialer Medien, von E‑Produkten, Computerspielen und Glücksspielen unter Auszubildenden.

**Methode:**

Querschnittliche Befragung von 4591 Auszubildenden an 17 beruflichen Schulen in Bayern, Schleswig-Holstein und Hamburg. Die Datenerhebungen mittels Fragebogen erfolgten zwischen März 2021 und April 2022. Die primären Endpunkte waren die 30-Tages-Prävalenz und das problematische Konsum- und Nutzungsverhalten der genannten Substanzen/Verhaltensweisen auf Basis von Screening-Instrumenten.

**Ergebnisse:**

Soziale Medien wurden mit einer 30-Tages-Prävalenz von 97,7 % am häufigsten von den Auszubildenden genutzt, gefolgt von Alkohol (64,3 %) und Computerspielen (55,8 %). Zigaretten wurden von 35,1 %, E‑Produkte von 17,9 % und Cannabis von 15,4 % konsumiert. Glücksspiele betrieben 12,2 % der Auszubildenden. Ein problematischer Konsum lag für Alkohol bei 47,4 %, für Tabak bei 18,0 %, für E‑Produkte bei 6,2 % und für Cannabis bei 1,6 % der Auszubildenden vor. Eine problematische Nutzung sozialer Medien wiesen 45,0 % der Auszubildenden auf, bei Glücksspiel waren 2,2 % und bei Computerspielen 0,7 % betroffen.

**Diskussion:**

Die Ergebnisse weisen darauf hin, dass es sich bei Auszubildenden um eine Risikogruppe für Suchtprobleme handelt, die erhöhten Interventionsbedarf aufweist. Vor allem Angebote der Sekundärprävention in den Bereichen Alkohol und soziale Medien sollten aufgrund ihrer weiten Verbreitung im Setting Berufsschule beachtet werden.

## Abstract (en)

**Background:**

The aim of the current study was to assess the prevalence of the (problematic) consumption of alcohol, tobacco, and cannabis as well as the (problematic) use of social media, e‑products, computer games, and gambling among apprentices.

**Method:**

Cross-sectional survey of 4591 apprentices at 17 vocational schools from Bavaria, Schleswig-Holstein, and Hamburg. Data was collected using questionnaires between March 2021 and April 2022. The primary endpoints were the 30-day prevalence and the problematic consumption and usage behavior of the mentioned substances/behaviors using screening instruments.

**Results:**

Among the assessed substances/behaviors, social media were used most frequently by the apprentices with a 30-day prevalence of 97.7%, followed by alcohol (64.3%) and computer games (55.8%). Cigarettes were consumed by 35.1%, e‑products by 17.9%, and cannabis by 15.4% of the apprentices. Of the apprentices, 12.2% reported having gambled in the past 30 days. Rates of problematic use were 47.4% for alcohol, 18.0% for tobacco, 6.2% for e‑products, and 1.6% for cannabis. Problematic use of social media was indicated by 45.0% of the apprentices, of gambling by 2.2%, and of computer games by 0.7%.

**Discussion:**

These results suggest that apprentices constitute a risk group for problematic substance use, indicating increased need for intervention. In particular, secondary prevention efforts in the areas of alcohol and social media should be taken into consideration due to their widespread prevalence in the vocational school setting.

## Hintergrund

Die Begrenzung von Schäden durch Substanzkonsum und andere abhängige Verhaltensweisen von Jugendlichen und jungen Erwachsenen ist ein wichtiges Anliegen der öffentlichen Gesundheit. Der Übergang von der Adoleszenz zum jungen Erwachsenenalter ist nicht nur mit einer Zunahme von riskantem und ungesundem Verhalten (wie z. B. Substanzkonsum) verbunden [1], sondern es hat sich auch gezeigt, dass früher Substanzkonsum die Wahrscheinlichkeit zur Entwicklung einer Substanzabhängigkeit erhöht [2].

Ein erheblicher Teil der Jugendlichen in Deutschland wechselt in der Adoleszenz vom allgemeinbildenden Schulsystem an die Berufsschule [3, 4]. Im Jahr 2022 lag die Anzahl der Auszubildenden in Deutschland bei rund 1,22 Mio. [5]. Der Übergang in die Berufsschule stellt für viele junge Menschen aufgrund der zunehmenden Selbstständigkeit und Eigenverantwortung eine Herausforderung dar. Ebenso scheint die Ausbildungszeit mit einer erhöhten Bereitschaft, gesundheitliche Risiken einzugehen, und insbesondere einem Anstieg des Substanzkonsums verbunden zu sein. Frühere Studien aus Deutschland zeigen, dass Auszubildende häufiger rauchen (61 % [6]; 62 % [7]) und Alkohol konsumieren (96 %; [6, 8]) als Gleichaltrige in der Allgemeinbevölkerung (30 % bzw. 74 %; [9]). Zudem identifizierte eine Befragung von über 5688 Auszubildenden einen riskanten Alkoholkonsum bei 45 % und eine Cannabisabhängigkeit bei 4 % der Auszubildenden [8]. Zur Verbreitung der Teilnahme an Glücksspielen und von glücksspielbezogenen Störungen sowie der problematischen Nutzung des Internets und von Computerspielen liegen bislang nur Daten aus einer Untersuchung unter Auszubildenden aus Mecklenburg-Vorpommern vor [7]. In dieser Studie wurde eine 12-Monats-Prävalenz des problematischen Betreibens von Glücksspielen von 10,6 % festgestellt; bei 2,1 % der Auszubildenden fanden sich Hinweise auf eine Störung durch das Betreiben von Glücksspielen nach DSM‑5. Gemäß der Compulsive Internet Use Scale (CIUS) gaben 16,6 % der Auszubildenden eine problematische Nutzung des Internets an und bei 6,2 % lagen Hinweise auf eine Internetabhängigkeit vor. Insgesamt 9,6 % der Auszubildenden berichteten problematisches Computerspielen und 3,4 % erfüllten die Kriterien einer Störung durch Spielen von Computerspielen im Internet nach DSM‑5. Ein im Vergleich zu Gleichaltrigen häufiger (risikoreicher) Substanzkonsum unter Auszubildenden wird weiter bestätigt durch Studien unter Auszubildenden aus der Schweiz [10, 11]. Einen risikoreichen Konsum von Alkohol berichteten 52 % [11] bzw. 62 % [10], von Cannabis 28 % der Auszubildenden. Zusätzlich wurde ein risikoreicher Internetgebrauch (gemäß der Compulsive Internet Use Scale) bei 75 % der Auszubildenden festgestellt [11].

Berufsschulen stellen ein wichtiges, aber bislang unterentwickeltes Setting für Maßnahmen der Prävention und Gesundheitsförderung dar. Um suchtpräventive Angebote zielgerichtet entwickeln und implementieren zu können, bedarf es Informationen zum (problematischen) Konsum- bzw. Nutzungsverhalten von Auszubildenden. Das Ziel der vorliegenden Arbeit ist es, die Verbreitung des (problematischen) Konsums von Alkohol, Tabak und Cannabis sowie die (problematische) Nutzung sozialer Medien, von Computerspielen und Glücksspielen in einer großen Stichprobe von Lernenden an beruflichen Schulen in Deutschland zu untersuchen. Zudem sollen Prädiktoren für die Verbreitung von (problematischem) Konsum- bzw. Nutzungsverhalten festgestellt und somit vulnerable Gruppen identifiziert werden, die bei der Gestaltung von Präventionsmaßnahmen besonders berücksichtigt werden sollten.

## Methoden

### Design

Datengrundlage dieser Arbeit bildet die Baseline-Befragung der Evaluationsstudie des Präventionsprogramms „Meine Zeit ohne – Die Challenge“ [12]. Die Studienpopulation bildeten Auszubildende an beruflichen Schulen in Bayern, Schleswig-Holstein und Hamburg. Die Gesamtstichprobe umfasste 4591 Auszubildende. Die Datenerhebung fand zwischen März 2021 und April 2022 statt. Insgesamt nahmen 277 Klassen aus 17 Schulen in Bayern (*n* = 5), Schleswig-Holstein (*n* = 7) und Hamburg (*n* = 5) an der Studie teil.

### Rekrutierung und Datenerhebung

In den 3 Bundesländern wurden bei den örtlichen Schulaufsichtsbehörden Genehmigungen für die Durchführung der Studie eingeholt. Die Berufsschulen wurden kontaktiert, über die Studie informiert und darum gebeten, bei Interesse an einer Teilnahme mit dem jeweiligen Studienzentrum in Kontakt zu treten. Die Mitarbeitenden der interessierten Schulen wurden mit Informationsmaterialien über den Zweck und den Ablauf der Studie sowie über den zeitlichen Aufwand aufgeklärt. Die Klassenlehrkräfte konnten anhand der vermittelten Informationen entscheiden, ob sie mit ihrer Klasse an der Studie teilnehmen. Bei einer Zusage wurde ein Zeitraum für die Datenerhebung festgelegt. Am Tag der Datenerhebung wurde während einer Unterrichtsstunde vor Ort das Projekt durch das Studienteam vorgestellt. Nach der Vorstellung des Projekts konnten die Auszubildenden entscheiden, ob sie an der Studie teilnehmen. Bei einer Zustimmung zur Teilnahme und der Einwilligung zur Datenverarbeitung füllten die Auszubildenden den Online-Fragebogen mit dem eigenen Handy, Tablet oder Laptop vor Ort aus. Den Auszubildenden, die kein eigenes technisches Endgerät besaßen, wurde durch das Studienteam für die Dauer der Befragung ein Tablet zur Verfügung gestellt. Während der gesamten Datenerhebung war das Studienpersonal vor Ort und unterstützte die Teilnehmenden bei Unklarheiten. In den Zeiten, in denen COVID-19-Verordnungen keinen Präsenzunterricht zuließen, fand die Projektvorstellung online statt. Für die Teilnahme gab es keine Ausschlusskriterien.

### Erhebungsinstrumente

Die Daten zum Konsum- und Nutzungsverhalten von Auszubildenden basierten auf Selbstauskünften und wurden anhand standardisierter Fragen erfasst. Zunächst wurden in den Bereichen Alkohol, Tabak, E‑Produkte, soziale Medien, Computerspiele und Glücksspiele der aktuelle Konsum- und Nutzungsstatus (Konsum/Nutzung in den letzten 30 Tagen: Ja/Nein) sowie die Anzahl der Tage, an denen dem jeweiligen Verhalten nachgegangen wurde, erhoben. Bezüglich des Spielens von Computerspielen wurde jede Form des Computerspielens, die auf einem Computer/Laptop, einer Gaming-Konsole oder auf einem anderen Gerät (z. B. Smartphone, Tablet) online und/oder offline stattgefunden hat, erhoben. Unter dem Betreiben von Glücksspielen wurde Toto, Lotto, Rubbellose, Bingo, Sportwetten, Spielautomaten mit Geldgewinnmöglichkeit, Poker, Casinospiele, Onlineglücksspiele oder Quizsendungen im Fernsehen verstanden. Beim Konsum von Cannabis wurde nur die 30-Tages-Prävalenz erhoben (Konsum: Ja/Nein). Zur Einstufung der Konsumintensität wurde die Anzahl der getrunkenen alkoholischen Getränke, die Anzahl gerauchter Zigaretten sowie die Häufigkeit der Nutzung von E‑Produkten an einem typischen Konsumtag erhoben. Zur Erfassung der Nutzungsintensität von Computerspielen und sozialen Medien wurden die Zeiten (Stunden und Minuten) erfasst, die an einem typischen Tag damit verbracht wurden; bzgl. sozialer Medien wurde zusätzlich erhoben, wie häufig am Tag auf sie zugegriffen wurde.

Zur Erhebung des *problematischen* Konsum- und Nutzungsverhaltens wurden validierte Fragebögen verwendet. Als problematischer Alkoholkonsum galt bei Frauen eine Punktzahl von ≥ 4 (Range = 0–12) und bei Männern und diversem Geschlecht eine Punktzahl von ≥ 5 im „Alcohol Use Disorder Identification Test – Consumption Items“ (AUDIT‑C; [13]). Ein problematischer Konsum von Zigaretten und E‑Produkten wurde bei einer Punktzahl von ≥ 3 (Range = 0–10) im „Fagerström Test for Nicotine Dependence“ (FTND; bei E‑Produkten in einer angepassten Version des FTND; [14]) definiert. Ein problematischer Cannabiskonsum lag bei einer Punktzahl von ≥ 7 (Range = 0–15) in der „Severity of Dependence Scale“ (SDS; [15, 16]) vor. Eine problematische Nutzung sozialer Medien wurde mit einer Punktzahl von ≥ 12 (Range = 6–30) in einer angepassten Version der „Bergen Facebook Addiction Scale“ (BFAS; [17, 18]) definiert, mit der auch andere soziale Medien erfasst wurden. Eine problematische Nutzung von Computerspielen lag bei einer Punktzahl von ≥ 32 (Range = 9–45) in der „Internet Gaming Disorder Scale – Short Form“ (IGDS-SF; [19, 20]) und problematisches Glückspiel bei einer Punktzahl von ≥ 1 (Range = 0–3) im „Brief Biosocial Gambling Screen“ (BBGS) vor [21].

Die erhobenen soziodemografischen Daten umfassten Geschlecht (männlich/weiblich/divers), Alter, Migrationshintergrund (beide Eltern in Deutschland geboren: Ja/Nein), Ausbildungszweig, Einkommen und Bildungsniveau. Eine ausführliche Beschreibung und Übersicht aller in der Studie erfassten Erhebungsinstrumente findet sich im Studienprotokoll [22].

### Statistische Auswertung

Die Beschreibung soziodemografischer Merkmale und des (problematischen) Konsum- und Nutzungsverhaltens der Auszubildenden erfolgte mit deskriptiven Statistiken (Prozent, Mittelwert, Standardabweichung). Zur Überprüfung von Zusammenhängen zwischen den untersuchten Variablen wurden Chi-Quadrat-Tests für Unabhängigkeit verwendet.

Zur Untersuchung möglicher Prädiktoren für das *problematische* Konsum- und Nutzungsverhalten (abhängige Variable: problematischer Konsum/problematische Nutzung: Ja/Nein) wurden für alle untersuchten Outcomes multiple logistische Regressionsanalysen gerechnet. Simultane Prädiktoren waren dabei: Geschlecht, Alter, Ausbildungszweig, Migrationshintergrund, monatliches Gehalt und Bildungsniveau. Für alle statistischen Analysen wurde ein Signifikanzniveau von *p* < 0,001 festgelegt. Alle Auswertungen wurden mit Stata 15.1 (StataCorp LLC, College Station, TX, USA; [23]) durchgeführt.

## Ergebnisse

### Stichprobe.

Von *n* = 4591 teilnehmenden Auszubildenden waren 53,9 % männlich, 45,2 % weiblich und 0,8 % divers. Das durchschnittliche Alter betrug 19,23 (*SD* = 4,20; *Range* = 15–55) Jahre und etwa ein Drittel (34 %) der Befragten wies einen Migrationshintergrund auf. Die monatlichen finanziellen Ressourcen lagen überwiegend (47,4 %) zwischen 600 € und 999 €. Die meisten Auszubildenden wiesen einen Mittel- oder Realschulabschluss (53,6 %) auf. Weitere soziodemografische Daten sind in Tab. 1 dargestellt.

**Table 1:** 

Variable	Ausprägung	Gesamt *n* = 4591
Geschlecht	Männlich	53,9 %
	Weiblich	45,2 %
	Divers	0,8 %
Alter, M (SD)	–	19,23 (4,20)
Migrationshintergrund	Nein	66,0 %
	Ja	34,0 %
Ausbildungsbereich	Schule/Ausbildungsvorbereitung	22,7 %
	Wirtschaft und Verwaltung	21,6 %
	Gewerbe und Technik	36,4 %
	Personenbezogene Dienstleistungen	19,4 %
Einkommen	Weniger als 600 €	37,8 %
	600–999 €	47,4 %
	1000 € und mehr	14,8 %
Bildungsniveau	Niedriger als Mittelschulabschluss	28,8 %
	Mittel- oder Realschulabschluss	53,6 %
	Allgemeine oder fachgebundene Hochschulreife	17,6 %

*M* Mittelwert, *n* Anzahl, *SD* Standardabweichung

### Prävalenzen des Konsums/der Nutzung.

Der Großteil der Auszubildenden (97,7 %) gab an, in den letzten 30 Tagen soziale Medien genutzt zu haben. Alkohol konsumierten etwa 2 Drittel aller Befragten (64,3 %). Mehr als die Hälfte der Auszubildenden (55,8 %) spielte Computerspiele, wobei der Anteil männlicher Auszubildender mehr als doppelt so hoch war wie der Anteil weiblicher Auszubildender (76,2 % bzw. 31,3 %). Etwa ein Drittel der Befragten gab an, in den letzten 30 Tagen Zigaretten geraucht zu haben (35,1 %). Nikotin in Form von E‑Produkten wurde von 17,9 % der Auszubildenden konsumiert und Cannabis von 15,4 %. Glücksspiele betrieben 12,2 % der Auszubildenden (18,3 % der männlichen und 4,9 % der weiblichen Auszubildenden; Tab. 2 und 3).

**Table 2:** 

		Alkohol		Zigaretten		E‑Produkte		Cannabis		Soziale Medien		Computerspiele		Glücksspiel	
Variable	*n*	30-Tages-Prävalenz	AUDIT-C ≥ 4/≥ 5	30-Tages-Prävalenz	FTND ≥ 3	30-Tages-Prävalenz	Adaptierter FTND ≥ 3	30-Tages-Prävalenz	SDS ≥ 7	30-Tages-Prävalenz	Adaptierter BFAS ≥ 12	30-Tages-Prävalenz	IGDS-SF ≥ 32	30-Tages-Prävalenz	BBGS ≥ 1
*Gesamt*	4591	64,3 %	47,4 %	35,1 %	18,0 %	17,9 %	6,2 %	15,4 %	1,6 %	97,7 %	45,0 %	55,8 %	0,7 %	12,2 %	2,2 %
*Geschlecht*															
Männlich	2476	67,1 %	46,6 %	37,0 %	19,6 %	17,7 %	6,3 %	17,5 %	1,7 %	97,4 %	32,4 %	76,2 %	0,9 %	18,3 %	3,5 %
Weiblich	2077	61,1 %	44,7 %	32,8 %	16,0 %	18,1 %	6,0 %	12,7 %	1,4 %	98,2 %	59,9 %	31,3 %	0,4 %	4,9 %	0,4 %
Divers	38	55,3 %	50,0 %	39,5 %	29,0 %	15,8 %	10,5 %	31,6 %	7,9 %	89,5 %	52,6 %	71,1 %	7,9 %	21,1 %	15,8 %
*Migrationshintergrund*															
Nein	3030	74,6 %	57,1 %	36,2 %	18,1 %	17,5 %	6,0 %	16,6 %	1,5 %	98,6 %	40,7 %	60,1 %	0,8 %	11,6 %	1,5 %
Ja	1561	44,2 %	28,6 %	33,0 %	17,8 %	18,6 %	6,5 %	13,1 %	1,9 %	96,0 %	53,4 %	47,5 %	0,6 %	13,5 %	3,5 %
*Ausbildungsbereich*															
Schule/Ausbildungsvorbereitung	992	50,3 %	36,0 %	28,2 %	12,9 %	15,2 %	5,7 %	16,4 %	2,2 %	96,5 %	47,6 %	60,3 %	0,6 %	10,3 %	2,2 %
Wirtschaft und Verwaltung	1041	60,8 %	42,1 %	37,0 %	19,8 %	18,7 %	6,4 %	16,7 %	1,8 %	98,6 %	50,0 %	44,8 %	1,1 %	9,8 %	1,6 %
Gewerbe und Technik	1669	76,6 %	60,5 %	38,2 %	19,1 %	18,0 %	5,9 %	14,4 %	1,2 %	97,8 %	33,2 %	69,9 %	0,7 %	18,2 %	3,1 %
Personenbezogene Dienstleistungen	889	60,7 %	42,0 %	34,8 %	19,6 %	19,6 %	7,0 %	14,6 %	1,6 %	97,8 %	58,5 %	37,2 %	0,6 %	6,1 %	1,1 %
*Einkommen*															
Weniger als 600 €	1736	54,0 %	38,4 %	30,7 %	15,6 %	16,1 %	6,2 %	15,6 %	1,8 %	96,8 %	48,0 %	57,1 %	0,6 %	9,7 %	2,3 %
600–999 €	2175	69,6 %	52,2 %	36,8 %	19,1 %	20,1 %	6,1 %	14,5 %	1,1 %	98,5 %	44,2 %	55,0 %	0,8 %	11,8 %	1,7 %
1000 € und mehr	680	73,4 %	55,0 %	40,9 %	20,9 %	15,3 %	6,5 %	17,9 %	2,8 %	97,2 %	39,9 %	55,2 %	0,7 %	20,0 %	3,5 %
*Bildungsniveau*															
Niedriger als Mittelschulabschluss	1323	50,4 %	36,6 %	38,9 %	24,2 %	19,7 %	8,2 %	15,9 %	2,0 %	94,4 %	45,8 %	54,4 %	1,2 %	9,8 %	2,2 %
Mittel- oder Realschulabschluss	2459	69,5 %	52,3 %	33,8 %	16,2 %	18,4 %	5,7 %	15,3 %	1,6 %	98,9 %	44,0 %	58,5 %	0,5 %	13,2 %	2,3 %
Hochschulreife	809	71,0 %	50,2 %	32,6 %	13,5 %	13,2 %	4,5 %	15,0 %	1,1 %	98,4 %	46,9 %	49,8 %	0,6 %	13,2 %	1,9 %

*AUDIT‑C* Alcohol Use Disorder Identification Test – Consumption Items, *BBGS* Brief Biosocial Gambling Screen, *BFAS* Bergen Facebook Addiction Scale, *FTND* Fagerström Test for Nicotine Dependence, *IGDS-SF* Internet Gaming Disorder Scale – Short Form, *n* Anzahl, *SDS* Severity of Dependence Scale

**Table 3:** 

Bereich	Konsum/Nutzung	Häufigkeit
Alkohol (*n* = 2951)	*Tage im Monat, M (SD)*	6,38 (5,87)
	*Audit‑C*	
	Risikoarmer Konsum (weiblich 0–3 Punkte, männlich und divers 0–4 Punkte)	29,1 %
	Riskanter Konsum (weiblich 4–12 Punkte, männlich und divers 5–12 Punkte)	70,9 %
	*Audit‑C Punktzahl (0–12), M (SD)*	5,81 (2,72)
	*Rauschtrinken in letzten 30 Tagen, Ja*	61,2 %
Zigaretten (*n* = 1611)	*Tage im Monat, M (SD)*	19,62 (11,89)
	*Zigaretten pro Tag, M (SD)*	7,67 (8,38)
	*FTND*	
	Geringe körperliche Abhängigkeit (0–2 Punkte)	48,7 %
	Mittlere körperliche Abhängigkeit (3–4 Punkte)	28,4 %
	Starke körperliche Abhängigkeit (5–6 Punkte)	17,3 %
	Sehr starke körperliche Abhängigkeit (7–10 Punkte)	5,7 %
	FTND-Punktzahl (0–10), M (*SD*)	3,27 (1,73)
Nikotinhaltige E‑Produkte (*n* = 820)	*Tage im Monat, M (SD)*	8,09 (9,39)
	*Nutzungshäufigkeit pro Tag, M (SD)*	6,70 (13,58)
	*Adaptierter FTND*	
	Geringe körperliche Abhängigkeit (0–2 Punkte)	65,5 %
	Mittlere körperliche Abhängigkeit (3–4 Punkte)	22,6 %
	Starke körperliche Abhängigkeit (5–6 Punkte)	8,2 %
	Sehr starke körperliche Abhängigkeit (7–10 Punkte)	3,8 %
	*FTND-Punktzahl (0–10), M (SD)*	2,75 (1,52)
Cannabis (*n* = 708)	*SDS*	
	Keine Abhängigkeit (0–6 Punkte)	89,4 %
	Abhängigkeit (7–15 Punkte)	10,6 %
	*SDS-Punktzahl (0–15), M (SD)*	2,48 (2,84)
Soziale Medien (*n* = 4485)	*Tage im Monat, M (SD)*	28,11 (5,73)
	*Minuten pro Tag, M (SD)*	235,74 (202,44)
	*BFAS*	
	Normale Nutzung (0–11 Punkte)	53,9 %
	Problematische Nutzung (12–18 Punkte)	36,3 %
	Abhängigkeit (19–30 Punkte)	9,8 %
	*BFAS-Punktzahl (6–30), M (SD)*	11,82 (4,72)
Computerspiele (*n* = 2562)	*Tage im Monat, M (SD)*	15,10 (10,59)
	*Minuten pro Tag, M (SD)*	178,53 (158,31)
	*IGDS-SF*	
	Keine Abhängigkeit (9–31 Punkte)	98,7 %
	Abhängigkeit (32–45 Punkte)	1,3 %
	*IGDS-SF Punktzahl (9–45), M (SD)*	16,92 (4,89)
Glücksspiel (*n* = 561)	*Tage im Monat, M (SD)*	4,14 (5,56)
	*BBGS*	
	Nicht-problematisches Glückspiel (0 Punkte)	82,2 %
	Problematisches Glückspiel (1–3 Punkte)	17,8 %
	*BBGS-Punktzahl (0–3), M (SD)*	0,29 (0,68)

*AUDIT‑C* Alcohol Use Disorder Identification Test – Consumption Items, *BBGS* Brief Biosocial Gambling Screen, *BFAS* Bergen Facebook Addiction Scale, *FTND* Fagerström Test for Nicotine Dependence, *IGDS-SF* Internet Gaming Disorder Scale – Short Form, *M* Mittelwert, *n* Anzahl, *SD* Standardabweichung, *SDS* Severity of Dependence Scale

### Problematischer Konsum/problematische Nutzung.

Fast die Hälfte der Auszubildenden zeigte einen problematischen Alkoholkonsum (47,4 %) und eine problematische Nutzung sozialer Medien (45,0 %). Die problematische Nutzung sozialer Medien weiblicher Auszubildender war fast doppelt so hoch wie die männlicher Auszubildender (59,9 % bzw. 32,4 %). Einen problematischen Nikotinkonsum in Form von Zigaretten (FTND-Wert ≥ 3) wiesen 18,0 % und in Form von E‑Produkten 6,2 % der Auszubildenden auf. Beim Betreiben von Glücksspielen zeigten 2,2 % und beim Spielen von Computerspielen 0,7 % der Befragten ein problematisches Nutzungsverhalten. Insgesamt 1,6 % der Gesamtpopulation wies einen problematischen Cannabiskonsum auf (Tab. 2).

### Prädiktoren des problematischen Konsums/der problematischen Nutzung.

Die Ergebnisse der logistischen Regressionsanalysen zeigten, dass das Geschlecht mit einer problematischen Nutzung von sozialen Medien und Glückspielen assoziiert ist: Frauen hatten im Vergleich zu Männern eine höhere Wahrscheinlichkeit für eine problematische Nutzung sozialer Medien. Hinsichtlich des Glücksspiels wiesen Frauen hingegen eine niedrigere Wahrscheinlichkeit für eine problematische Nutzung auf. Das Alter war mit dem problematischen Konsum von Alkohol und Zigaretten negativ assoziiert: Jüngere Auszubildende hatten eine höhere Wahrscheinlichkeit für einen problematischen Alkoholkonsum als ältere Auszubildende. Mit steigendem Alter stieg jedoch die Wahrscheinlichkeit für einen problematischen Zigarettenkonsum.

Auszubildende mit Migrationshintergrund wiesen im Vergleich zu Auszubildenden ohne Migrationshintergrund eine geringere Wahrscheinlichkeit für einen problematischen Alkoholkonsum auf. Eine höhere Wahrscheinlichkeit zeigte sich hingegen für eine problematische Nutzung von sozialen Medien und von Glücksspielen.

Auch die Ausbildungsrichtung war mit dem problematischen Konsum von Alkohol assoziiert: Bei Auszubildenden aus dem Ausbildungszweig „Gewerbe und Technik“ wurde eine höhere Wahrscheinlichkeit für einen problematischen Konsum von Alkohol festgestellt als bei Auszubildenden in der schulischen Ausbildung bzw. der Ausbildungsvorbereitung.

Die Analysen zeigen, dass mit einem höheren Einkommen der problematische Konsum von Alkohol steigt. Im Vergleich zu Auszubildenden, die weniger als 600 € verdienten, zeigten Auszubildende mit einem Einkommen von „600–999 €“ und „1000 € und mehr“ eine höhere Wahrscheinlichkeit für einen problematischen Alkoholkonsum.

Ein ähnlicher Trend wie bei Einkommen und Alkohol zeigte sich auch beim Bildungsniveau: Hier korrespondierte die Wahrscheinlichkeit des Konsums von Alkohol mit einer höheren Bildung. So wurde bei Auszubildenden mit einem Mittel‑/Realschulabschluss sowie mit einer Hochschulreife eine höhere Wahrscheinlichkeit, Alkohol problematisch zu konsumieren, festgestellt als bei Auszubildenden mit einem Haupt- oder Förderschulabschluss. Zigarettenkonsum war hingegen negativ mit dem Bildungsniveau assoziiert: Bei Auszubildenden mit einem Mittel‑/Realschulabschluss sowie mit einer Hochschulreife war die Wahrscheinlichkeit für einen problematischen Zigarettenkonsum niedriger als bei Auszubildenden mit einem Haupt- oder Förderschulabschluss. Für mehr Informationen siehe Tab. 4.

**Table 4:** 

	Alkohol	Zigaretten	E‑Produkte^a^	Cannabis	Soziale Medien	Computerspiele	Glücksspiel
Variable	OR 95 %-KI	OR 95 %-KI	OR 95 %-KI	OR 95 %-KI	OR 95 %-KI	OR 95 %-KI	OR 95 %-KI
**Geschlecht**							
Männlich (Ref.)	–	–	–	–	–	–	–
Weiblich	1,15 1,00–1,34	0,73** 0,61–0,88	0,89 0,67–1,19	0,66 0,39–1,13	2,72*** 2,36–3,13	0,41* 0,17–0,97	0,10*** 0,05–0,22
Divers	1,33 0,67–2,66	1,34 0,61–2,92	1,64 0,56–4,76	3,94* 1,12–13,89	2,52** 1,30–4,89	9,39** 2,55–34,64	4,16** 1,50–11,49
Alter	0,96*** 0,94–0,98	1,06*** 1,04–1,08	1,01 0,98–1,04	1,00 0,95–1,05	0,97** 0,96–0,99	0,93 0,81–1,06	1,03* 1,00–1,07
**Migrationshintergrund**							
Nein (Ref.)	–	–	–	–	–	–	–
Ja	0,33*** 0,29–0,38	0,86 0,73–1,01	1,01 0,78–1,31	1,15 0,71–1,86	1,65*** 1,44–1,88	0,77 0,36–1,65	2,55*** 1,69–3,85
**Ausbildungsbereich**							
Schule/Ausbildungsvorbereitung (Ref.)	–	–	–	–	–	–	–
Wirtschaft und Verwaltung	1,04 0,83–1,29	1,59** 1,20–2,10	1,25 0,82–1,91	1,05 0,51–2,17	0,96 0,78–1,19	2,23 0,69–7,18	1,27 0,62–2,61
Gewerbe und Technik	**2**,06*** 1,68–2,53	1,25 0,96–1,63	1,03 0,69–1,54	0,52 0,25–1,07	0,82 0,67–1,00	0,94 0,30–2,97	1,26 0,68–2,33
Personenbezogene Dienstleistungen	1,16 0,94–1,44	1,53** 1,16–2,01	1,28 0,85–1,94	0,91 0,43–1,90	1,19 0,96–1,46	1,25 0,35–4,46	1,23 0,55–2,76
**Einkommen**							
Weniger als 600 € (Ref.)	–	–	–	–	–	–	–
600–999 €	1,46*** 1,24–1,72	1,27* 1,04–1,55	1,05 0,77–1,44	0,80 0,42–1,49	0,97 0,82–1,14	1,53 0,62–3,77	0,64 0,37–1,11
1000 € und mehr	1,85*** 1,49–2,30	1,34* 1,03–1,73	1,19 0,79–1,79	2,06* 1,05–4,04	0,86 0,69–1,07	1,45 0,45–4,72	1,07 0,58–1,99
**Bildungsniveau**							
Niedriger als Mittelschulabschluss (Ref.)	–	–	–	–	–	–	–
Mittel- oder Realschulabschluss	1,56*** 1,35–1,81	0,57*** 0,48–0,67	0,68** 0,52–0,89	0,90 0,54–1,50	1,04 0,90–1,20	0,46* 0,21–0,98	1,28 0,80–2,06
Hochschulreife	1,55*** 1,25–1,90	0,32*** 0,25–0,42	0,48** 0,31–0,73	0,52 0,23–1,18	1,16 0,94–1,42	0,50 0,16–1,56	0,97 0,48–1,93

*KI* Konfidenzintervall, *OR* Odds Ratio, *Ref.* Referenzkategorie

*p < 0,05; **p < 0,01; ***p < 0,001; *%* Prozent

^a^nur nikotinhaltige E‑Produkte

## Diskussion

Die vorliegende Studie untersuchte das (problematische) Konsum- und Nutzungsverhalten von Auszubildenden und liefert somit eine aktuelle Datenlage zur Verbreitung des (problematischen) Konsums von Alkohol, Tabak/E-Produkten und Cannabis sowie der Nutzung sozialer Medien, von Computerspielen und Glücksspielen bei Auszubildenden in Deutschland.

### Substanzkonsum

Der (problematische) Substanzkonsum unter Auszubildenden war über die untersuchten Substanzen hinweg hoch und teilweise weiter verbreitet als in der Gesamtpopulation dieser Altersgruppe. Verglichen mit den Daten der 18- bis 25-Jährigen in der Drogenaffinitätsstudie aus dem Jahr 2021 [24] lag bei den Auszubildenden in der vorliegenden Studie eine niedrigere 30-Tage-Prävalenz des Alkoholkonsums (71,5 % vs. 64,3 %) mit einem deutlich höheren Anteil problematischer Konsumentinnen und Konsumenten vor (16,8 % vs. 47,4 %). In einer früheren Untersuchung unter Auszubildenden wurden ähnlich hohe Anteile festgestellt (Alkoholkonsum: 68,9 %; problematischer Alkoholkonsum: 45 %; [8]). Die 30-Tage-Prävalenz des Zigarettenkonsums (28,9 % vs. 35,1 %) und der tägliche Zigarettenkonsum (14,7 % vs. 16,8 %) der Auszubildenden in unserer Studie lagen leicht über dem von jungen Erwachsenen in der Allgemeinbevölkerung [24]. In früheren Studien unter Auszubildenden aus den Jahren 2013 und 2015 wurde ein höherer täglicher Zigarettenkonsum berichtet (40,7 % [8] und 56,5 % [6]). Dies dürfte auf den allgemein abnehmenden Trend des Zigarettenkonsums zurückzuführen sein, der auch bei Auszubildenden zum Tragen kam [25]. Der Anteil der Nutzerinnen und Nutzer von E‑Produkten unter Auszubildenden lag unter dem der jungen Erwachsenen in der Allgemeinbevölkerung (24,4 % vs. 17,9 %; [24]). Zur problematischen Nutzung von E‑Produkten liegen leider keine Vergleichsdaten vor. Die Daten der vorliegenden Studie weisen auf eine geringe Verbreitung eines problematischen Cannabiskonsums hin (1,6 %). Die Prävalenz des Konsums von Cannabis in den letzten 30 Tagen lag jedoch mit 15,4 % leicht über der Jugendlicher und junger Erwachsener in der Allgemeinbevölkerung (12,0 %; [24]). Zum problematischen Cannabiskonsum liegen uns keine geeigneten Konsumdaten aus der gleichaltrigen Allgemeinbevölkerung vor.

### Problematische Nutzung sozialer Medien, von Computerspielen und Glücksspielen

In der vorliegenden Studie konnte eine weite Verbreitung der Nutzung sozialer Medien (97,7 %) und von Computerspielen (55,8 %) festgestellt werden. Während der Anteil problematischer Nutzung von Computerspielen unter Auszubildenden mit 0,7 % gering war, ist er bei sozialen Medien (45 %) als kritisch zu betrachten. Die Daten der Drogenaffinitätsstudie aus dem Jahr 2019 zeigten bei 21,9 % der 18- bis 25-Jährigen eine problematische Nutzung und bei 5,5 % eine Computerspiel-/internetbezogene Störung (gemäß des CIUS; [26]).

In dieser Studie gaben 12,2 % der Auszubildenden an, in den letzten 30 Tagen Glücksspiel betrieben zu haben. In einer Repräsentativbefragung im Jahr 2019 ergab sich eine 12-Monats-Prävalenz der Teilnahme an irgendeinem Glücksspiel von 23,3 % bei 18- bis 20-Jährigen und von 29 % bei 21- bis 25-Jährigen in der Allgemeinbevölkerung [27]. Der Anteil der befragten Auszubildenden mit einem problematischen Glücksspiel lag in unserer Studie bei 2,2 %. Da keine geeigneten Daten aus der gleichaltrigen Allgemeinbevölkerung zum problematischen Glücksspiel vorliegen [28], bleibt unklar, ob Auszubildende weniger oder mehr durch die Teilnahme an Glücksspielen belastet sind als andere Gleichaltrige.

### Implikationen

Das hohe Risiko eines problematischen Konsum- und Nutzungsverhaltens unter Auszubildenden unterstreicht die Bedeutung von Berufsschulen als Setting für Präventionsmaßnahmen. Bei Präventionsmaßnahmen für Auszubildende muss jedoch zum einen die größere Heterogenität bezüglich des Alters und des Bildungsniveaus [11] im Vergleich zum Klassenverband in allgemeinbildenden Schulen beachtet werden. Zum anderen wird durch das duale System an Berufsschulen ein anderer Rahmen vorgegeben, in den Präventionsmaßnahmen eingebettet werden müssen. Während Schülerinnen und Schüler in Klassen allgemeinbildender Schulen meist die gesamte Schulzeit gemeinsam verbringen, verbringen Auszubildende ihre Zeit sowohl in der Berufsschule als auch im Betrieb. Diese Zeitaufteilung könnte einen Einfluss auf die klassen- bzw. schulbezogenen Sozialisationseffekte, also auf die Dynamik unter den Auszubildenden, haben. Diese Dynamik spielt jedoch in Präventionsprogrammen für das allgemeinbildende Schulsystem oft eine große Rolle (z. B. im Programm „Be smart, don’t start“). Es stellt sich jedoch die Frage nach der Passung solcher empirisch evaluierter Interventionsprogramme, die sich mehrheitlich an eine jüngere Zielgruppe richten und für das Setting der allgemeinbildenden Schule entwickelt wurden. Obwohl sich berufliche Schulen bemühen, Suchtpräventionsprogramme anzubieten, handelt es sich hierbei meist um nichtevaluierte Maßnahmen. Für das Setting Berufsschule fehlt es an systematischen Angeboten, die flächendeckend eingesetzt werden können [29].

Bei der Entwicklung von effektiven Präventionsprogrammen sollten neben dem Setting 2 weitere Aspekte besondere Beachtung finden: Zum einen die jeweilige Substanz oder Verhaltensweise, deren Konsum bzw. Nutzung vorgebeugt werden soll, zum anderen sollten vor allem vulnerable Gruppen angesprochen und erreicht werden. Im Berufsschulsetting wird aufgrund der hohen Werte für problematischen Konsum bzw. problematische Nutzung in den Bereichen Alkohol und soziale Medien besonderer Interventionsbedarf gesehen. In Übereinstimmung mit der Literatur wiesen in unserer Studie jüngere Auszubildende im Vergleich zu älteren einen höheren problematischen Alkoholkonsum auf [8, 11, 30]. Wie eine frühere Untersuchung bestätigt [8], scheint auch eine höhere Bildung mit einem problematischen Alkoholkonsum in Zusammenhang zu stehen. So war bei Auszubildenden mit einem Mittel‑/Realschulabschluss sowie mit einer Hochschulreife die Wahrscheinlichkeit für einen problematischen Konsum von Alkohol höher als bei Auszubildenden mit einem Haupt- oder Förderschulabschluss. In unserer Studie hing auch das Einkommen mit einem problematischen Alkoholkonsum positiv zusammen. Ebenso erwies sich das Vorliegen eines Migrationshintergrunds in unserer Studie als ein Schutzfaktor für problematischen Alkoholkonsum [8, 10]. Präventionsangebote zur Vorbeugung des Alkoholkonsums sollten insbesondere jüngere Auszubildende erreichen sowie den Bildungsabschluss und das Einkommen der Auszubildenden berücksichtigen.

Unsere Ergebnisse zeigen weiterhin, dass die problematische Nutzung sozialer Medien mit dem Geschlecht und dem Vorliegen eines Migrationshintergrunds assoziiert sind. Die häufigere problematische Nutzung sozialer Medien von weiblichen Auszubildenden im Vergleich zu männlichen zeigte dabei die gleiche Tendenz wie in einer Untersuchung unter Auszubildenden in der Schweiz [11]. Auch das Vorliegen eines Migrationshintergrunds fungierte als Risikofaktor einer problematischen Nutzung sozialer Medien. Auszubildende mit einem Migrationshintergrund zeigten deutlich häufiger eine problematische Nutzung sozialer Medien als Auszubildende ohne Migrationshintergrund. Vor allem weibliche Auszubildende und solche mit Migrationshintergrund sollten deshalb mit Maßnahmen zur Prävention einer problematischen Nutzung sozialer Medien angesprochen werden.

### Limitationen

Bei der Interpretation der Daten sind folgende Limitationen zu beachten. Es handelt sich bei der vorliegenden Stichprobe nicht um eine repräsentative Stichprobe, jedoch um eine große Gelegenheitsstichprobe von Schülerinnen und Schülern an beruflichen Schulen aus 3 Regionen, die bereit waren an einer randomisiert-kontrollierten Studie zur Ermittlung der Wirksamkeit des Präventionsprogramms „Meine Zeit ohne – Die Challenge“ teilzunehmen [12]. Auch konnten in der Untersuchung nicht alle Ausbildungszweige berücksichtigt werden. Die Ergebnisse können daher nicht auf die Allgemeinheit der Auszubildenden übertragen werden. Die in dieser Studie ermittelten Hinweise, dass Auszubildende im Vergleich zu Gleichaltrigen höhere Konsumrisiken haben könnten, sollte im Rahmen von weiteren, repräsentativen Studien geprüft werden.

Bei der Betrachtung der Ergebnisse muss auch der Zeitraum der Datenerhebung zwischen März 2021 und April 2022 berücksichtigt werden, in dem teilweise noch Beschränkungen aufgrund der COVID-19-Pandemie bestanden, die das Konsum- und Nutzungsverhalten der Auszubildenden beeinflusst haben könnten. In der Literatur finden sich Hinweise sowohl auf eine Zunahme als auch auf eine Abnahme des Konsums während der COVID-19-Pandemie (z. B. [31, 32]). Konsumdaten von Auszubildenden während der Pandemie liegen jedoch nicht vor. Zudem lässt die querschnittliche Erhebung der Daten keine Aussagen hinsichtlich kausaler Zusammenhänge zu.

## Fazit

Die Ergebnisse der vorliegenden Studie liefern aktuelle Daten zum Konsum- und Nutzungsverhalten von Auszubildenden in Deutschland. Sie unterstreichen die Annahme, dass es sich bei Auszubildenden um eine Risikogruppe für Suchtprobleme handelt, die erhöhten Interventionsbedarf aufweist. Dieser erfordert die Entwicklung von evidenzbasierten Präventionsangeboten für diese Zielgruppe, da bestehende Angebote, die für das Setting der allgemeinbildenden Schulen entwickelt wurden, meist nicht direkt auf das Setting der Berufsschule übertragen werden können. Aufgrund der weiten Verbreitung des problematischen Alkoholkonsums und der problematischen Nutzung sozialer Medien sind vor allem Maßnahmen der Sekundärprävention zu empfehlen, die diese Bereiche einschließen. Zudem sollten vulnerable Gruppen bei der Ansprache besonders berücksichtigt werden.
